# Clinical significance of CDX2-positive circulating tumour cells in colorectal cancer patients

**DOI:** 10.1038/bjc.2011.32

**Published:** 2011-03-01

**Authors:** S C C Wong, S S M Ng, M T Cheung, L Y Luk, C M L Chan, A H K Cheung, V H M Lee, P B S Lai, B B Y Ma, E P Hui, M Y Y Lam, T C C Au, A T C Chan

**Affiliations:** 1 State Key Laboratory in Oncology in South China, Department of Clinical Oncology, Sir YK Pao Centre for Cancer, Hong Kong Cancer Institute and Prince of Wales Hospital, The Chinese University of Hong Kong, Hong Kong Special Administrative Region, China; 2 Department of Surgery, Prince of Wales Hospital, The Chinese University of Hong Kong, Hong Kong Special Administrative Region, China; 3 Department of Surgery, Queen Elizabeth Hospital, Hong Kong Special Administrative Region, China

**Keywords:** clinical significance, CDX2-positive circulating tumour cells, colorectal cancer

## Abstract

**Background:**

Our recent work has shown the feasibility of using a refined immunomagnetic enrichment (IE) assay to detect cytokeratin 20-positive circulating tumour cells (CK20 pCTCs) in colorectal cancer (CRC) patients. We attempted to improve the sensitivity for CRC by detecting another intestinal-type differentiation marker, CDX2 pCTCs, using the same methodology.

**Methods:**

CDX2 pCTCs were detected in patients with CRC, colorectal adenoma (CAD), benign colorectal diseases (BCD), other common cancers (OCC) and normal subjects (NS). Statistical analysis was used to correlate CDX2 pCTCs to the clinicohistopathological factors, recurrence, metastasis and survival after follow-up for 42 months in CRC patients.

**Results:**

CDX2 pCTCs were detected in 81% CRC patients (73 out of 90, median number=21.5 CTCs), 7.5% CAD patients (3 out of 40), 0% patients with BCD (0 out of 90), 2.5% patients with OCC (2 out of 80) and 0% NS (0 out of 40). Furthermore, statistical analysis showed that CDX2 pCTC numbers were associated with tumour- node-metastasis stage and lymph node status. Using the median CDX2 pCTC numbers as the cutoff points, stratified groups of CRC patients had significant differences in their recurrence and survival.

**Conclusions:**

This study showed that the refined IE assay can detect CDX2 pCTCs with high sensitivity and that CDX2 pCTCs can generate clinically important information for CRC patients.

The identification of circulating tumour cells (CTCs) can be used to detect malignancy, predict metastasis, evaluate prognosis, assist in the management of cancer patients and monitor recurrence and metastasis after primary therapy (Braun and Naume, 2005; Mocellin *et al*, 2006; Paterlini-Brechot and Benali, 2007; Sleijfer *et al*, 2007). Approaches to detect CTCs can be classified into molecular-based methods, which detect target mRNA expression, and cytometric methods, which isolate and quantify individual cells (Paterlini-Brechot and Benali, 2007). Although researchers have reached an important consensus that cytopathological examination of CTCs after immunomagnetic enrichment (IE), with further characterisation of their malignant potential, represents a promising approach (Cristofanilli *et al*, 2004, 2006), the current IE method uses either a broad-spectrum anti-cytokeratin (CK) antibody (Miltenyi Biotec, Bergisch Gladbach, Germany), combined anti-CK8, anti-CK18 and anti-CK19 antibodies (CellSearch system, Veridex, Warren, NJ, USA) or an anti-BerEP_4_ antibody (Dynal Biotech, Invitrogen, Carlsbad, CA, USA) against general epithelial antigens of various tumour and normal cells. Therefore, specific information on the primary tumour type is not available. Our recent work has overcome this limitation by blocking the Fc region of the anti-BerEP_4_ antibody with a goat anti-mouse antibody during IE, so that an anti-CK20 antibody can be used to show the gastrointestinal (GI) origin of the BerEP_4_-positive cells in the blood of colorectal cancer (CRC) patients (Wong *et al*, 2009). This modification can improve immunomagnetic CTC detection by allowing tumour- or tissue-specific antibodies to bind to their respective antigens so that an accurate diagnosis of the tumour type can be made. As the detection rate of CK20-positive circulating tumour cells (CK20 pCTCs) in CRC was only 62% (Wong *et al*, 2009), we aimed to improve the sensitivity for CRC by detecting another intestinal-type differentiation marker, CDX2 pCTCs, using the same methodology. CDX2 has critical functions in intestinal development, differentiation and maintenance of the intestinal phenotype (Takakura *et al*, 2010). Moreover, CDX2 is a more specific marker than CK, CK8, CK18, CK19 and BerEP_4_ because the former antigen is expressed mainly in tumour and normal cells from the GI tract, whereas the latter antigens are expressed in virtually all carcinomas and all non-neoplastic epithelial cells (Latza *et al*, 1990; Chu and Weiss, 2002). Therefore, we expect that CDX2 pCTCs may more accurately reflect the micrometastatic condition of the CRC patients. In this study, the refined assay was used to evaluate the clinical significance of CDX2 pCTCs in CRC by detecting such cells in patients with CRC, colorectal adenoma (CAD), benign colorectal diseases (BCD) and other common cancers (OCC). We further correlated CDX2 pCTC numbers to the clinicohistopathological factors, recurrence, metastasis and survival after follow-up for 42 months. The information obtained in this study would be very useful for understanding the prognostic and diagnostic potential of CDX2 pCTCs in CRC patients.

## Materials and methods

### Blood samples of patients

Between March 2003 and August 2007, blood samples were taken from three cohorts of patients. In the first cohort, blood samples from 90 CRC patients and 40 CAD patients were taken at two time points: (1) before operation (stages I–III CRC patients on the basis of tumour node metastasis (TNM) classification), before therapeutic intervention (TNM stage IV CRC patients) and before endoscopy (CAD patients) and (2) on first follow-up (7 days after operation for CRC patients and 7 days after endoscopy for CAD patients). Moreover, 64 TNM stages I–III CRC patients were followed up for 42 months from their respective diagnosis for recurrent or metastatic CRC and the disease-free survival (DFS) in patients with (1) negative preoperative CDX2 pCTC, (2) positive preoperative CDX2 pCTCs and decreased postoperative level and (3) positive preoperative CDX2 pCTCs and increased postoperative level, were compared separately. Furthermore, overall survival (OS) in 90 TNM stages I–IV CRC patients using the median number of pretreatment CDX2 pCTC as the cutoff point was studied. As the TNM stage IV patients in our cohort did not receive curative treatment, the OS of 64 TNM stages I–III CRC patients using the median number of preoperative CDX2 pCTC as the cutoff point was also compared. In the second cohort, blood samples from 30 patients each with colitis, haemorrhoids, colorectal ulcers and hyperplastic polyps were taken before surgical treatment. In the third cohort, blood samples from 20 patients each with breast cancer, prostate cancer, liver cancer and lung cancer were taken before operation or treatment. We evaluated all CRC patients preoperatively by routinely performing a computerised tomography (CT) in the abdomen and pelvis before surgery in order to rule out liver metastasis, peritoneal deposits or lymphadenopathy. In addition, positron emission tomography (PET) scan was performed in CRC patients when there was an uncertainty in metastasis. In patients with increased postoperative CDX2 pCTCs, CT and PET scans were used to search for residual disease. Finally, 40 normal subjects (NS) were also recruited for comparison. Although the NS did not undergo colonoscopy to confirm their status, their plasma samples had been tested for carcinoembryonic antigen (CEA) protein and all were within the normal range. Informed consent was obtained from all patients and healthy individuals. The clinicohistopathological characteristics of the studied subjects were shown in Table 1. The study was approved by the Clinical Research Ethics Committee of the Prince of Wales Hospital and Queen Elizabeth Hospital, Hong Kong Special Administrative Region.

### Refined IE assay, immunocytochemical (ICC) staining and examination of CDX2 pCTCs in blood samples

Ten millilitres of blood from each sample was collected in EDTA tubes. The mononuclear cells were collected by centrifugation through a Ficoll density gradient (catalogue no. 17-1440-02; GE Healthcare, Uppsala, Sweden), according to the manufacturer's instructions. The CTCs were isolated from the mononuclear cells using the refined protocol as shown in our recent study (Wong *et al*, 2009) and CDX2 ICC staining was performed for each patient sample. The criteria used to identify CDX2 pCTCs in a blood sample were as follows: (1) positive CDX2 staining, (2) the cell should have a round-to-oval morphology and (3) the cell size should be at least double that of a lymphocyte. The CDX2 pCTCs were examined and quantified by two independent assessors and an average cell number was calculated for each patient sample.

### Statistical analysis

Multivariate regression was used to analyse whether preoperative CDX2 pCTCs were correlated with the clinicohistopathological factors of the patients and Cox's proportional hazards model was applied to detect the independent prognostic factors of survival (Statistical Package for the Social Sciences Version 12.0 software, SPSS Inc., Chicago, IL, USA). Besides, *χ*^2^ test was used to examine the association between preoperative CDX2 pCTCs and recurrent or metastatic CRC. Kaplan–Meier method was used to plot the OS and DFS curves and log-rank test was used to examine whether the OS and DFS of selected patient groups stratified by the median of preoperative CDX2 pCTCs had significant difference (GraphPad Prism software version 4.0, GraphPad, Software Inc., San Diego, CA, USA). A *P*-value <0.05 was considered to be statistically significant.

## Results

### CDX2 pCTCs in patients with CRC, CAD and NS

**Figure 1:**
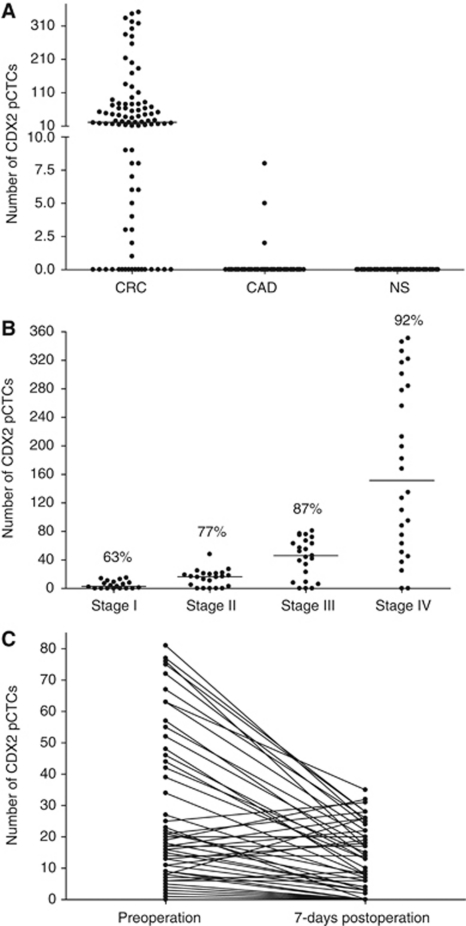
CDX2 pCTCs in blood samples. (**A**) Number of CDX2 pCTCs per 10-ml blood sample in 90 CRC patients (preoperation), 40 CAD patients (preendoscopy) and 40 NS. (**B**) Number of CDX2 pCTCs per 10-ml blood sample in 19 stage I, 22 stage II, 23 stage III and 26 stage IV CRC patients. (**C**) Number of CDX2 pCTCs per 10-ml blood sample in 64 CRC patients (preoperation and first follow-up) with interconnecting lines between the two time points. The median in each group of subjects is indicated by a black horizontal line.

**Figure 2:**
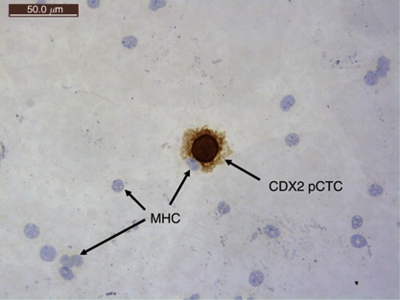
A typical CDX2 pCTC from a CRC patient sample. MHC: mononuclear hematopoietic cell (original magnification, × 400).

CDX2 pCTC cannot be found in any of the 40 NS (Figure 1A); therefore, the baseline was set at 0 CTC and detection of ⩾1 CTC per 10-ml blood was considered to be positive. Detailed analysis showed that the overall detection rate in CRC, CAD patients and NS were 81% (73 out of 90, range: 0–351), 7.5% (3 out of 40, range: 0–8) and 0% (0 out of 40), respectively (Figure 1A). When we divided the CRC patients into different TNM stages, the detection rates were 63% (12 out of 19, stage I), 77% (17 out of 22, stage II), 87% (20 out of 23, stage III) and 92% (24 out of 26, stage IV) (Figure 1B). On their first follow-up, only 64 CRC patients (stages I–III) were recruited because 26 patients with stage IV did not undergo surgery. Among them, 49 patients (49 out of 64=77%) had detectable preoperative CDX2 pCTCs and 35 of 49 patients (71%) were found to have a decreased number of postoperative CDX2 pCTCs, whereas 9 of 49 (18%) patients had increased number of postoperative CDX2 pCTCs (Figure 1C). In contrast, only three CAD patients had detectable preendoscopy CDX2 pCTCs and none of them showed any CDX2 pCTCs after endoscopy. A typical CDX2 pCTC from a CRC patient was shown in Figure 2.

### Multivariate regression analysis

Multivariate regression analysis was applied to examine whether pretreatment CDX2 pCTCs was correlated with the clinicohistopathological factors of the 90 TNM stages I–IV CRC patients. Significant associations were found with TNM stage (*P*<0.001) and lymph node status (*P*<0.01) but not for age (*P*=0.672), sex (*P*=0.854), tumour stage (*P*=0.385) and degree of differentiation (*P*=0.316).

### Recurrent or metastatic CRC

The median number of preoperative CDX2 pCTCs from the 64 TNM stages I–III CRC patients was 13.5. Using this median number as the cutoff point, 10 patients with preoperative CDX2 pCTCs >13.5 and only three patients with preoperative CDX2 pCTCs ⩽13.5 had recurrent or metastatic CRC after follow-up for 42 months and the association between preoperative CDX2 pCTCs and recurrent or metastatic CRC was highly significant (*χ*^2^ test: *χ*^2^=4.73; *P*<0.05).

### Survival of CRC patients

**Figure 3:**
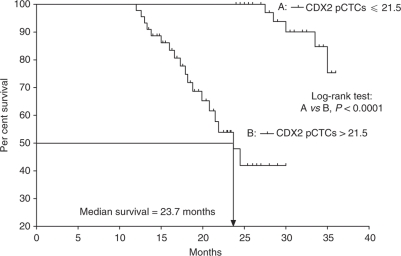
Overall survival analysis for 45 CRC patients with (A) CDX2 pCTCs ⩽21.5 and (B) 45 CRC patients with CDX2 pCTCs >21.5.

**Figure 4:**
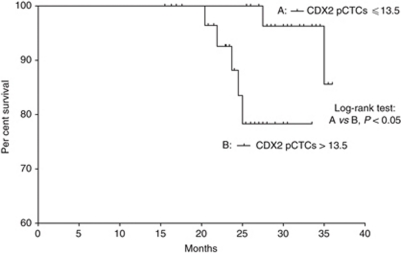
Overall survival analysis for 45 CRC patients with (A) CDX2 pCTCs ⩽13.5 and (B) 45 CRC patients with CDX2 pCTCs >13.5.

**Figure 5:**
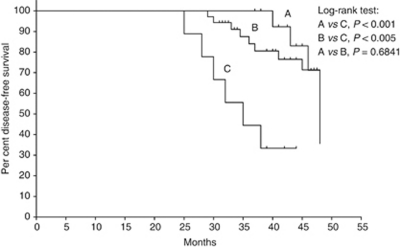
Disease-free survival analysis in CRC patients with (A) negative preoperative CDX2 pCTC, (B) positive preoperative CDX2 pCTCs and decreased postoperative level and (C) positive preoperative CDX2 pCTCs and increased postoperative level.

Overall survival curves were plotted for patients with pretreatment CDX2 pCTCs >21.5 and those with pretreatment CDX2 pCTCs ⩽21.5, where 21.5 is the median number of pretreatment CDX2 pCTCs from the first cohort of 90 CRC patients. Our results showed that the survival rates for those two groups of patients were significantly different (*P*<0.0001, log-rank test; Figure 3). Moreover, the independent prognostic factors of OS identified by the Cox's proportional hazards regression model were found to be pretreatment CDX2 pCTCs (*P*=0.003) and lymph node status (*P*=0.022; Table 2). Furthermore, the OS in stages I–III patients stratified by the median number of preoperative CDX2 pCTCs of 13.5 had significant difference (*P*<0.05, log-rank test; Figure 4). Among the nine patients who had increased postoperative number of CDX2 pCTCs, two were stage III and seven were stage II. Follow-up for 42 months showed that one stage III patient and two stage II patients died of disease, whereas one stage III patient and two stage II patients had disease progression of either recurrence or metastasis. Stratifying the patients into subgroups indicated significant differences in DFS between (a) patients with negative preoperative CDX2 pCTCs and those with positive preoperative CDX2 pCTCs and increased postoperative level (*P*<0.001, log-rank test; Figure 5) and (b) patients with positive preoperative CDX2 pCTCs and decreased postoperative level and those with positive preoperative CDX2 pCTCs and increased postoperative level (*P*<0.005, log-rank test; Figure 5).

### CDX2 pCTCs in patients with BCD and OCC

Pretreatment CDX2 pCTCs can be detected in none of the patients with BCD (colitis, haemorrhoids, colorectal ulcers and hyperplastic polyps) and 2.5% (2 out of 80) patients with other OCC (breast cancer, prostate cancer, liver cancer and lung cancer).

## Discussion

Although the clinical significance of CTCs from patients with tumours is still debatable (Giribaldi *et al*, 2006; Katsumata *et al*, 2006), the technology platform has improved rapidly. Over the last few years, CTC detection has become more standardised and reliable (Naoe *et al*, 2007; Riethdorf *et al*, 2007). A typical example is the detection of CTCs with the CellSearch System, which allows the defined stratification of the risk of death in metastatic breast cancer patients (Cristofanilli *et al*, 2005). However, the anti-CK antibody panel (CK8, CK18 and CK19) in this system is not specific in tumour typing. Therefore, we hypothesise that the detection of a specific marker in CTCs with quantification might be helpful in the prognosis and diagnosis of CRC patients.

The success of the refined IE assay to detect CK20 pCTCs in CRC patients has opened up a new scenario in the detection of CTCs (Wong *et al*, 2009). Although the sensitivity of CK20 pCTCs for CRC was only 62%, other more sensitive and specific markers can be explored using this refined IE assay. In this study, CDX2 pCTC was chosen to be examined with three objectives because CDX2 is both a sensitive and specific marker of intestinal differentiation and it is overexpressed in CRC tumour cells when compared with normal intestinal epithelium (Witek *et al*, 2005). The first objective is to evaluate (1) the sensitivity of preoperative CDX2 pCTCs for CRC and CAD, (2) the origin of those CDX2 pCTCs and (3) the prognostic potential of preoperative CDX2 pCTCs. Our results show that the overall detection rate in CRC was 81% and detailed analysis indicated that the detection rates are higher for stages III and IV CRC, whereas the detection rates are lower for stages I and II CRC. These results are logical because the dissemination of tumour cells into blood is a micrometastatic process, which has a higher metastatic potential in stages III and IV tumours than in stages I and II tumours (Payne, 1989). Moreover, our observation is supported by a previous report, which showed that the CTCs are the metastatic precursors with an increased malignant potential when compared with the parental cells in the primary tumour (Glinskii *et al*, 2003). On the other hand, preendoscopy CDX2 pCTCs can only be found in three CAD patients with severe dysplasia; this low percentage (7.5%) is expected because CAD is a pre-malignant lesion. In summary, our results suggest that the presence of CDX2 pCTCs may be a late event in colorectal carcinogenesis.

At 7 days after operation, CDX2 pCTC numbers were found to be decreased in 67% (8 out 12) stage I, 47% (8 out of 17) stage II and 95% (19 out of 20) stage III CRC patients. This finding provides evidence that the origin of those preoperative CDX2 pCTCs is the primary tumour. Therefore, we suggest that CDX2 pCTC may be a better CRC biomarker than serum CEA because a persistent high serum CEA level after surgery can be explained by many reasons. The possible causes include overlooked metastases or inadequate surgery (Duffy, 2001). However, smoking habits, renal insufficiency, chronic pulmonary or liver diseases and pancreatitis, as well as postoperative complications such as mechanical bowel obstruction caused by surgery may also contribute to this condition (Duffy, 2001; Wang *et al*, 2007). Therefore, patients with high CEA concentration after surgery should be thoroughly studied with the understanding that elevated CEA often, but not always, predicts the recurrence of CRC (Filiz *et al*, 2009). A rise in postoperative CEA levels before clinically observable recurrence was reported in 18–75% of cases with CRC relapse (Moertel *et al*, 1993). The survival analysis indicated that there is no significant difference between patients with known and unknown causes of high CEA levels; however, patients whose CEA successfully returned to normal levels clearly showed a better survival rate (Filiz *et al*, 2009). Collectively, these findings suggest that early detection and operation would reduce CDX2 pCTCs, which may diminish the risk of metastasis to other distant organs. It is interesting to note that nine patients had an increase in CDX2 pCTCs after surgery for 7 days. We hypothesise that this phenomenon may be due to the presence of residual tumour after surgery. Follow-up of all TNM stage I–III CRC patients for 42 months showed that patients with increased postoperative CDX2 pCTC numbers had the worst DFS when compared with those with decreased postoperative CDX2 pCTC numbers and those with negative preoperative CDX pCTC. Therefore, CDX2 pCTCs may be useful to select CRC patients with high risk of recurrence. Moreover, our data provide evidence that CDX2 pCTC is a potential marker to show the effectiveness of surgical resection or other local treatment modalities, and a larger scale study to compare CDX2 pCTC with a conventional CRC marker, such as serum CEA, for validation of this important function will be carried out. Overall, the detection rate of CDX2 pCTCs in various TNM stages of CRC patients using this refined assay (81%) was higher than that using CellSearch System in CRC patients by Sastre *et al* (2008) (36.2%). This discrepancy can be explained by the fact that Sastre *et al* (2008) performed blood collection after surgery in TNM stage I–III CRC patients and that most of them had partial or complete clearance of CTCs (Sastre *et al*, 2008). In fact, the percentage of CDX2 pCTCs detected in our cohort of CRC patients at their first follow-up after operation was only 62.5% (40 out of 64). Another reason that can explain this difference in CRC patients is that Sastre *et al* (2008) used ⩾2 CTCs rather than ⩾1 CTC in that study as the cutoff point for positivity. In CAD patients, three patients with severe dysplasia who had two, five and eight CDX2 pCTCs, respectively, before endoscopy showed no CDX2 pCTCs after endoscopy, which suggests that the origin of those CDX2 pCTCs is the adenoma lesion. Our results support previous reports that indicate that some CAD tissue specimens already have cancer cell clones with unfavourable histology (Bertario *et al*, 2003; Church, 2004; Jin *et al*, 2007). Therefore, early removal can prevent them from changing into a malignant lesion later. Finally, the absence of CDX2 pCTC in all 40 healthy subjects suggests that this assay has a low false-positive rate.

The prognostic potential of CDX2 pCTCs is shown by its significant correlation to TNM stage, lymph node status, recurrence or metastasis. These results are expected because during tumour growth, primary tumour cells will continuously shed into the blood circulation and the lymphatic system. A small number of tumour cells in the blood or the lymph may be able to survive and metastasise to distant organs, such as liver or lung. In addition, more tumour cells from TNM stages III and IV CRC patients will shed into the blood and the lymph than those from TNM stages I and II CRC patients because of the higher proliferation rate in the advanced stage primary tumour (Valera *et al*, 2005). Using the median CDX2 pCTC numbers of 21.5 as the cutoff point, pretreatment CDX2 pCTCs correlated with OS in CRC patients. Actually, the patients with CDX2 pCTCs >21.5 were mainly of stages III and IV, whereas those with CDX2 pCTCs ⩽21.5 were mostly of stages I and II. This may be one explanation why stages III and IV patients have a greater risk of recurrence, metastasis and shorter survival than stages I and II patients. Excluding stage IV CRC patients, the significant difference in OS using the median CDX2 pCTC numbers of 13.5 shows that preoperative CDX2 pCTC may predict survival. This finding, together with the result that CRC patients with increasing postoperative CDX2 has a worse DFS, shows that a portion of CRC patients in our cohort, with both higher preoperative and postoperative CDX2, had the worst OS and DFS.

In the second objective, we did not observe any pretreatment CDX2 pCTC in patients with various kinds of BCD. This finding can imply that those BCD do not have micrometastatic potential. In fact, the significance of pretreatment CDX2 pCTC in CRC detection would be greatly reduced if it were found in patients with BCD. Therefore, we propose that patients with benign diseases should be included in all tumour marker evaluation studies in order to have a comprehensive assessment of the potential of the markers in prognosis and diagnosis.

In the third objective, we explored whether CDX2 pCTCs can be found in other cancers because currently one major limitation in IE CTCs detection is that only broad-spectrum antibodies are used and therefore detection is not specific to any kind of tumour or tissue system. Previous studies indicated that IE CTCs detection using broad spectrum antibody is very promising only in metastatic breast cancer (Cristofanilli *et al*, 2004, 2005, 2006), whereas there are still very scanty reports regarding CRC (Molnar *et al*, 2001; Allard *et al*, 2004; Sastre *et al*, 2008). Using this refined IE assay with standardised ICC staining and stringent assessment criteria, CDX2 pCTC was only found in two prostate cancer patients. This finding confirms previous studies that reported CDX2 expression in prostate adenocarcinoma (Herawi *et al*, 2007; Leite *et al*, 2008). In summary, our results are encouraging because it can prove that this refined IE assay in CDX2 pCTCs detection is rather specific to CRC among the types of cancers that have been tested.

To the best of our knowledge, this study is the first to detect CDX2 pCTCs in CRC patients, using a GI-specific anti-CDX2 antibody. According to the American Society of Clinical Oncology (ASCO) 2006 update of recommendations for the use of tumour markers in GI cancer, serum CEA test can be ordered preoperatively if it would assist in staging and surgical planning (Locker *et al*, 2006). Postoperative CEA levels should also be assessed every 3 months for stages II and III of the disease for at least 3 years if the patient is a potential candidate for surgery or chemotherapy of metastatic disease (Locker *et al*, 2006). Moreover, CEA is the marker of choice for monitoring the response of metastatic disease to systemic therapy (Locker *et al*, 2006). On the other hand, the ASCO 2006 guidelines also admit that serum CEA test has insufficient sensitivity for detecting primary and recurrent CRC and this test may be useful as a first-line surveillance investigation in CRC patients during surgical follow-up based on serial CEA measurements, using temporal trends in conjunction with clinical, radiological and/or histological confirmation (Locker *et al*, 2006; Tan *et al*, 2009; Yamashita and Watanabe, 2009). Furthermore, the ASCO guidelines comment that another CRC marker, carbohydrate antigen 19.9, suffers from low sensitivity and specificity in CRC and it has been proven ineffective as screening, diagnostic and prognostic tools (Locker *et al*, 2006; Tan *et al*, 2009; Yamashita and Watanabe, 2009). Therefore, it is necessary to develop novel biomarkers for CRC detection and monitoring. Quantification of CDX2 pCTCs, as detected by this refined IE assay, has high potential for the differential diagnosis of CRC and their serial measurements may be clinically useful to monitor disease progression.

Finally, the success of this refined IE assay has opened up new possibilities in the detection of CTCs as the shedding CTCs from various cancers may be further characterised after IE with their respective specific tumour markers, using ICC staining (Wong *et al*, 2004), *in situ* hybridisation (Wong *et al*, 2002) or even molecular profiling using quantum dot technology (Gao and Nie, 2003; Smith *et al*, 2006).

## Figures and Tables

**Table 1:** Clinicohistopathological characteristics of studied subjects

*Colorectal carcinoma patients (n=90)*	
Sex	
Male	59 (66%)
Female	31 (34%)

Age (years*)*	
Range	26–91
Median	67

TNM classificati*on*	
Tumour stage	
T1	14 (16%)
T2	26 (29%)
T3	29 (32%)
T4	21 (23%)
Lymph node status	
N0	41 (46%)
N1	23 (25%)
N2	26 (29%)

TNM stage	
Stage I	19 (21%)
Stage II	22 (24%)
Stage III	23 (26%)
Stage IV	26 (29%)

Degree in differentiation	
Well	15 (17%)
Moderate	51 (57%)
Poor	24 (26%)
	
*Colorectal adenoma patients (n=40)*	
Sex	
Male	22 (55%)
Female	18 (45%)

Age (years)	
Range	26–78
Median	53

Degree in dysplasia	
Mild	6 (15%)
Moderate	23 (58%)
Severe	11 (27%)
	
*Apparently normal subjects (n=40)*	
Sex	
Male	19 (48%)
Female	21 (52%)
Age (years)	
Range	22–70
Median	34

Abbreviation: TNM=tumour node metastasis.

**Table 2:** Multivariate regression for overall survival by Cox's proportional hazards regression

**Parameter**	***P*-value**	**Relative hazard**	**95% CI for relative hazard**
Preoperative CDX2 pCTCs (>21.5 and ⩽21.5)	0.003 (S)	9.274	4.372–12.795
Sex (male *vs* female)	0.651 (NS)	—	—
Age (>71 *vs* ⩽71 years)	0.949 (NS)	—	—
pT category (T1+T2 *vs* T3+T4)	0.752 (NS)	—	—
Differentiation (well *vs* poor)	0.816 (NS)	—	—
Lymph node involvement (presence *vs* absence)	0.022 (S)	6.293	2.749–9.271

Abbreviations: CI=confidence interval; NS=non-significant; pCTC=positive circulating tumour cell; S=significant; TNM=tumour node metastasis.
